# Increased Brain Conductivities in Patients with Alzheimer’s Disease

**DOI:** 10.1002/alz.085500

**Published:** 2025-01-09

**Authors:** Geon‐Ho Jahng, Hak Young Rhee, Soonchan Park, Chang‐Woo Ryu

**Affiliations:** ^1^ Kyung Hee University Hospital at Gangdong, Seoul Korea, Republic of (South)

## Abstract

**Background:**

The levels of conductivity in the extracellular and intracellular space of the brain are still unknown, especially in the brains of Alzheimer’s disease (AD) patients. The objectives of this study were to investigate how the extra‐neurite conductivity (EC) and intra‐neurite conductivity (IC) were reflected in AD patients compared with elderly CN people and patients with amnestic mild cognitive impairment (MCI) and to evaluate the association between EC and cognitive decline.

**Method:**

A total of 66 patients were included including 20 AD patients, 25 amnestic mild cognitive impairment (MCI) patients, and 21 cognitively normal (CN) old people. For the brain magnetic resonance electrical property tomography (MREPT) images, a multi‐echo turbo spin‐echo pulse sequence was used. High‐frequency conductivity (HFC) at the Larmor frequency was calculated and decomposed into extra‐ (EC) and intra‐neurite (IC) conductivities, respectively. Each parameter map was compared among the three participant groups using the full factorial design of one‐way analysis of covariance (ANCOVA). Furthermore, the association between the value of each map and either age or the MMSE score, with age as a covariate, was evaluated using the multiple regression analysis.

**Result:**

The conductivities of both HFC and EC were increased in the AD group in the large brain areas. The IC was increased in AD compared with CN but decreased in AD compared with MCI. Compared with the CN group, all three conductivity indices were increased in the MCI group. EC was higher in the AD group in the hippocampus (p=0.009) and insular (p=0.020). Both HFC and EC were significantly negatively associated with the MMSE in the insular and MTG, but IC were significantly positively associated with the MMSE in the corpus callosum.

**Conclusion:**

The EC value was higher in patients with AD than CN elderly people and patients with amnestic MCI. The EC value decreased with increasing MMSE scores after adjusting for age in the insula and middle temporal gyrus. This study showed the possibility that the EC value might be used as an imaging biomarker for helping to monitor cognitive function.

